# Implementing a prebrief for cultural humility in standardized patient sessions with genetic counseling students

**DOI:** 10.1002/jgc4.70098

**Published:** 2025-08-29

**Authors:** Teresa Chai, Lisa Jay Kessler, Janice Radway, Denise LaMarra, Kathleen Valverde

**Affiliations:** ^1^ Master of Science in Genetic Counseling Program, Perelman School of Medicine University of Pennsylvania Philadelphia Pennsylvania USA; ^2^ Standardized Patient Program, Perelman School of Medicine University of Pennsylvania Philadelphia Pennsylvania USA

**Keywords:** communication skills, cultural humility, genetic counseling education, prebrief

## Abstract

Standardized patient (SP) sessions allow students to practice cultural humility and gain confidence in providing care for diverse patient populations. A prebrief (Pb) occurs before participation in the SP session and involves three steps: planning, briefing, and facilitating. Prebriefing is effective in integrating cultural humility in healthcare education fields, such as nursing. Similar data are not yet available for the genetic counseling field. This article describes one genetic counseling program's experience piloting the use of a Pb before a genetic counseling SP session centered around cultural humility. Thirty‐five learners from two different cohorts of the University of Pennsylvania Master of Science in Genetic Counseling Program participated in this SP session with a Pb and were invited to participate in the study. Learner skill use and cultural humility were assessed, and feedback on the Pb was collected. Descriptive statistics were used to analyze the data. The increase in learner counseling skill use and scores on Foronda's Cultural Humility Scale are outlined. Also discussed are logistics around this pilot's creation, implementation, and future directions. It was concluded that a Pb promotes discussion and reflection before the SP session and can aid in genetic counseling students' education, cultural humility, and interpersonal skill use.

**What is known about this topic?**

Prebriefs (Pbs) have been used as an educational tool in various healthcare fields, including nursing, occupational therapy, and more recently, genetic counseling. Using a Pb before standardized patient sessions has been shown to maximize learning, decrease student anxiety, and increase student knowledge on cultural differences.

**What does this paper add to the topic?**

Using a Pb in genetic counseling to incorporate and emphasize cultural humility has yet to be described. This paper adds one way a Pb can be implemented in genetic counseling education with simulations focused on cultural humility. It also highlights student perspectives on using a Pb in genetic counseling and perceived benefits and limitations of a Pb in genetic counseling standardized patient sessions. Furthermore, it exemplifies how a Pb before a genetic counseling standardized patient session can assist learners with cultural humility, skill use, and overall learning.

## 1 INTRODUCTION

Standardized patient (SP) methodology, or human simulation, is used in healthcare education, including nursing, occupational therapy, physical therapy, and genetic counseling (GC). SPs are laypersons trained to portray patients and family members in a consistent, measurable manner to teach and assess students and to provide practice for emotionally and medically challenging cases (Kessler et al., 2021). Following the Accreditation Council for Genetic Counseling (ACGC) standards, GC programs include SP sessions, allowing students opportunities to practice and refine clinical and interpersonal skills (ACGC, 2023; Kessler et al., 2021). Student use of more patient‐centered communication skills in an SP session is associated with higher SP satisfaction ratings (Lowe & Roter, 2022). These ratings can then be used to assess GC student performance and could be extrapolated into actual patient satisfaction.

In addition to teaching communication skills, SP sessions can allow learners to practice cultural humility (Kourgiantakis et al., 2020; Qin & Chaimongkol, 2021; Ward‐Gaines et al., 2021). Cultural humility is an openness toward self‐reflection about our existence as culturally embedded beings and a willingness to strive to understand the backgrounds and identities of others to avoid categorizing and overgeneralizing their experiences (Hook et al., 2016; Kessler & Love, 2020). Prasad et al. assert that cultural humility is a “foundation for students to consider possible power imbalances.… when cultural differences may have an impact on the potential clinical outcome (Prasad et al., 2016).” Addressing culture, power, and bias with SPs can be sensitive; learners may benefit from additional preparation (Foronda et al., 2022).

Foronda et al. suggest using a prebrief (Pb) for cultural humility to address bias with healthcare trainees. In accordance with the Healthcare Simulation Standards of Best Practice, SP sessions should include creation of a “prebriefing plan that includes preparation materials and briefing to guide participant success in the simulation‐based experience” (Rossler et al., 2021). Educators lead the Pb to prepare learners for the SP session. The Pb is tailored to the learner's level of training and the learning objectives of the case. Using a Pb for a cultural humility‐based SP session with nurse practitioner students increased student knowledge of cultural differences, enhanced critical thinking skills, and satisfaction (Ndiwane et al., 2017).

There is limited research about Pbs and genetic counseling. Jacobs and McEwen report the use of a Pb in genetic counseling and that most learners were satisfied with its use (Jacobs & McEwen, 2024). However, there is currently no research about using a Pb for cultural humility in genetic counseling. This pilot study implemented a Pb before a GC SP session centered around cultural humility. We report on our experience, potential ways to evaluate student outcomes (cultural humility and skill use) from the Pb and SP session, and the benefits and limitations of such an implementation.

## 2 SP CASE AND METHODS

**FIGURE 1:**
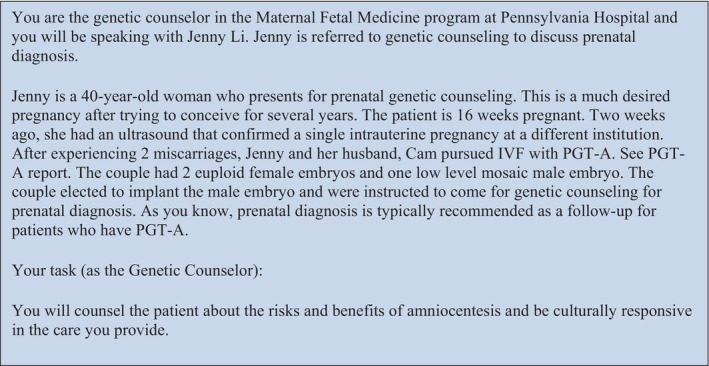
Doorway instructions.

Learners received a GC scenario case that is more common in Eastern cultures. Learners more accustomed to Western cultures may be unfamiliar with or have differing viewpoints on this cultural scenario. Figure 1 outlines the case and instructions for learners before the encounter and Pb. Additional information about the case development and SP training can be found in the Appendix S1.

### 2.1 Prebrief

During the Pb, learners watched a 5‐minute video providing cultural context and significance behind the case. The video was developed to emphasize the literature available on sex selection and prenatal genetic counseling, including perspectives of genetic counselors with Eastern cultural backgrounds (Bowman‐Smart et al., 2020; Tsai et al., 2017). Following the video, learners had time to discuss and ask questions. The SPs received training, including the same Pb video the learners watched. The Pbs were approximately 25 min.

### 2.2 Participants

In April 2023 and 2024, all second‐year students at the University of Pennsylvania Master of Science in Genetic Counseling Program (UPenn MSGC) were invited via email to participate (*n* = 17 in 2023, *n* = 18 in 2024). Students who declined to participate in the study still participated in the Pb and GC session described above as part of their education in the program. However, they were not sent any study surveys. Participants were surveyed at two different time points: before participation in the Pb and following completion of the SP session. All participants completed the same SP case and Pb. In 2023, 16 students (94%) completed the preliminary survey regarding cultural humility. After completing the SP session with Pb, all consented students were sent a second set of surveys addressing cultural humility and their experience with the Pb. Thirteen responses (81%) were collected. In 2024, 15 students participated (83%), and 9 (60%) completed the second round of surveys. A comparison group consisted of 16 former UPenn MSGC second‐year students from a previous graduating class who participated in an SP session of a similar case without a Pb component.

### 2.3 Instrumentation and data analysis

Participants completed two surveys. Foronda's Cultural Humility scale uses 19 items to evaluate cultural humility across three domains: context for the difference in perspective, self‐attributes, and outcomes of cultural humility (Foronda et al., 2021). Participants completed this survey before the Pb and again after completing the SP session. An interpretation guide scores participants into one of four categories: rarely culturally humble, sometimes culturally humble, usually culturally humble, and habitually culturally humble. Following the SP session with a Pb, participants were given the Prebrief Experience Survey (PES), adapted from Reed's Debriefing Experience Scale (Reed, 2012). The PES has five categories: Analyzing Thoughts and Feelings, Learning and Making Connections, Facilitator Skill in Conducting the Prebriefing, Appropriate Facilitator Guidance, and Additional Feedback. It includes 18 items on a 5‐point Likert‐type scale that ranges from strongly agree to strongly disagree as well as three open‐ended questions asking for detailed feedback on the Pb experience.

**FIGURE 2:**
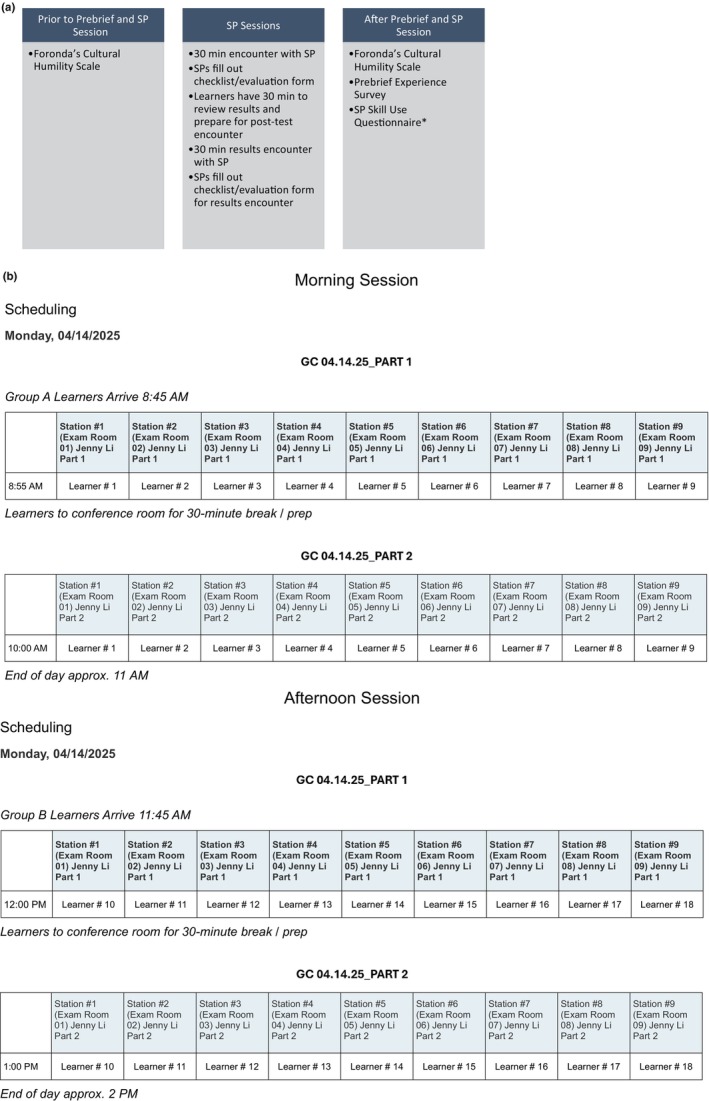
(a) Timing and instruments used in the study. *Survey completed by authors not study participants. (b) Student schedules for SP sessions.

Additionally, the MSGC program and SP faculty developed checklists for SPs to assess learners on their objectives and interpersonal skills. The authors used the participants' SP evaluation and feedback forms to complete the SP Skill Use Questionnaire (Lowe & Roter, 2022). This consisted of a 21‐item measure to assess which skills the GC learners used in the session. Skills were divided into five domains: LISTEN (eliciting patient questions and concerns), EDUCATE (information exchange), ASSESS (facilitating adherence), PARTNER (shared decision‐making), and SUPPORT (interpersonal rapport). These skill domains overlap with many ACGC PBCs. Figure 2, illustrates the surveys' learners completed and the timing of the SP sessions. REDCap was utilized to distribute the surveys and record all data (Harris et al., 2009, 2019). Descriptive statistics were used to analyze participants' responses, and one‐tailed *t*‐tests were used to compare learners who participated in the case with a Pb and those who participated in a similar case without a Pb. The study was reviewed and granted an exemption by the University of Pennsylvania review board (#853258).

## 3 RESULTS

Although there were no statistically significant differences between the group with a Pb and the group without a Pb, descriptively, there was an increase in student skill use and cultural humility. When comparing skill use among learners who participated in the SP session with a Pb to previous learners who participated in a similar SP case without a Pb, learners with a Pb used more skills across all domains (LISTEN, EDUCATE, ASSESS, PARTNER) than learners without a Pb. This was noted particularly in the ASSESS domain, with 13% of learners without a Pb using the skill compared to 85% (2023) and 89% (2024) of learners who completed a Pb. Table 1 outlines the skills examined and examples of questions for evaluating the domains. More participants also scored in the habitually culturally humble category following the case completion in 2023 and 2024 (*p* = 0.5) (Table 2).

**TABLE 1:** Skill use domains, evaluation questions, and learner skill use percentages with and without a prebrief.

Skill use domain	Skill use prompt	SP evaluation form question	% learners who used skill‐ No Prebrief group (*n* = 16)	%learners who used skill—Prebrief group 2023 (*n* = 13)	%learners who used skill—Prebrief group 2024 (n=9)
LISTEN (eliciting patient questions and concerns)	The GC asked my opinion about treatment and testing options	Did the student explain amniocentesis? Ensure the patient wanted the information?	75	85	100
EDUCATE (information exchange)	The GC involved me in discussing and setting the visit agenda	Did the student give you an opportunity to collaboratively set goals for the appointment?	88	100	100
ASSESS (facilitating adherence)	The GC asked nonjudgmental questions about my lifestyle	Did the student acknowledge the importance of the baby's sex to the patient and try to learn more about why it is so important?	13	85	89
PARTNER (shared‐decision making)	The GC helped me consider risks, benefits, and options	Did the student use scenario building?	100	100	100

**TABLE 2:** Foronda's Cultural Humility Scale pre‐ and post‐SP session student scores.

Foronda's cultural humility scale score	# 2023 students pre‐SP session (*n* = 13)	# 2023 students post‐SP session (*n* = 13)	# 2024 students pre‐SP session (*n* = 9)	# 2024 students post‐SP session (*n* = 9)
Rarely culturally humble	0	0	0	0
Sometimes culturally humble	1	0	1	0
Habitually culturally humble	10	6	5	5
Habitually culturally humble	2	7	3	4

*Note*: Signficance for pre‐SP vs post‐SP in 2023 was 0.092. Significance for pre‐SP vs post‐SP in 2024 was 0.57.

Overall, participants reported positive learning outcomes from the Pb. Most learners agreed that the Pb helped them to analyze their thoughts and feelings and make connections and that the facilitator appropriately guided them in the Pb. The complete Prebrief Experience Survey and participants' average responses are found in Table 3. There were no responses of “strongly disagree” for any of the questions, and only one participant disagreed with any statements on the PES. In the open‐ended feedback, participants appreciated having more context behind the case and time to reflect on the various psychosocial and cultural differences. One participant noted that the “Pb provides context and prepares us for a psychosocially more involved case. It helped me confront my biases about cultural perceptions of gender equality, which I had become aware of previously in the clinic but still needed to think through.”

**TABLE 3:** Prebrief experience survey and average response.

Category	Prompt	Average response
Analyzing thoughts and feelings	1. Prebriefing helped me to analyze my thoughts about the SP case	Agree
	2. The prebriefing environment was physically comfortable and psychologically safe	Agree
Learning and making connections	1. Prebriefing helped me to make connections in my learning	Agree
	2. Prebriefing helped me to make connections between theory and real‐life situations	Agree
	3. Prebriefing was helpful in processing the context of the SP case	Agree
	4. Prebriefing provided me with a learning opportunity	Agree
	5. Prebriefing helped me to find meaning in the SP session	Neither agree nor disagree
	6. My questions and problems about the SP session prompt were answered by prebriefing	Agree
	7. Prebriefing helped me to make connections between theory and real‐life situations	Agree
	8. I became more aware of my own cultural biases during the prebriefing session	Neither agree nor disagree
	9. The prebrief helped me to understand how someone's cultural background may influence their thoughts and decisions	Agree
Facilitator skill in conducting the prebrief	1. The facilitator allowed me enough time to verbalize my feelings before commenting	Agree
	2. The prebriefing session facilitator talked the right amount during prebriefing	Agree
	3. Prebriefing provided a means for me to reflect on my thoughts before the SP session	Agree
	4. I had enough time to prebrief thoroughly	Agree
	5. The prebriefing session facilitator was an expert in the content area	Agree
Appropriate facilitator guidance	1. The facilitator taught the right amount during the prebriefing session	Agree
	2. The facilitator provided adequate guidance during the prebriefing	Agree

Abbreviation: SP, standardized patient.

Conversely, one participant reported that having more context upfront made the session feel more simulated and was unrealistic compared to what one would expect as a practicing genetic counselor. “If the goal of the SP session is to simulate a real patient session, the Pb takes away from that a little bit. You usually don't go into a session with this much knowledge about cultural context/patients' reasons for decision making, etc. It may facilitate student learning, but it is less ‘realistic’ this way.”

## 4 DISCUSSION

This case demonstrated the potential value of using a Pb for cultural humility before a GC SP session. When presimulation activities and discussions, similar to Pbs, were applied in other healthcare settings with culturally based simulations, learners were found to improve in terms of knowledge, self‐confidence, and critical thinking (Ndiwane et al., 2017). Although there were no statistically significant differences between groups, descriptively, more skills were used by participants in this case with a Pb compared to a cohort of second‐year learners at the same time point in training who participated in a similar case without a Pb, indicating that the Pb can facilitate increased skill use among learners. A key objective of the Pb and SP session was to help learners develop the skills to facilitate conversations around unfamiliar cultural scenarios. The ASSESS domain evaluated whether learners acknowledged and inquired further about the cultural context during the SP session. Learners who participated in a Pb demonstrated a greater improvement in this domain than was seen in learners who did not, underscoring the effectiveness of the Pb. Many learners provided positive feedback about the Pb, noting how it gave additional context behind the case and more time to process their thoughts.

The use of a Pb has recently been described in genetic counseling (Jacobs & McEwen, 2024). However, this study's Pb introduced an SP session and focused on creating a co‐counseling plan as learners participated in the simulation in pairs. Learners in the described study felt the Pb was too short in duration; however, in the current study, all learners either agreed or strongly agreed that they had adequate time in the Pb (Table 3). The Jacob and McEwen study described the Pb use during the first week of the learners' graduate education. In contrast, the Pb in this study was introduced at the end of the final year of the learner's education. These differences in learner education and goals of the Pb (co‐counseling vs. cultural humility) may have contributed to the differing opinions on adequate Pb time. Both studies reported high learner satisfaction with using a Pb before a GC SP session. Further discussion about the feasibility of implementing a Pb for a GC SP session can be found in the Appendix S1.

Using a Pb to help develop learners' cultural humility is a relatively new concept, and measures to evaluate the outcomes of this application of a Pb are still being discussed (Foronda et al., 2021). The balance between giving learners more context and time to discuss the case and preserving the authenticity of the simulation has yet to be explored. If the goal of the SP session is to facilitate learning and attain the ACGC practice‐based guidelines and accreditation standards, the results and learner feedback would suggest that a Pb before a GC SP session can assist learners with cultural humility, skill use, and overall learning.

### 4.1 Limitations and future directions

A major limitation of this pilot study was the small sample size, which reduced the statistical power and limits the generalizability of our experience. Since Foronda's Cultural Humility Scale was given twice, it is also possible that some of the post‐SP session responses were influenced by seeing the questions in the pre‐SP session survey. Additionally, as a program‐developed evaluation form was used to fill out the SP Skill Use Questionnaire instead of directly giving the SPs the questionnaire to fill out, it is possible that we did not fully capture student skill use and potential changes in skill use from the Pb. In the future, it may be helpful to directly use the questionnaire and conduct a fully randomized study comparing students who get the same exact cultural case, with half receiving a Pb and the other half not receiving a Pb. Our experience provides one way in which a Pb can be developed and used with cases centered around cultural humility. This is the first time that a Pb has been used by the Penn MSGC program. It would be important to see if similar Pbs can be developed and implemented for other GC SP cases centered around cultural humility.

## 5 CONCLUSION

Introducing a focused Pb on cultural humility before an SP session represents a new educational model in GC. Promoting discussion and reflection before the SP session can aid in GC education by supporting the development of cultural humility, skill use, and can be applied to real‐life situations, including culturally complex situations, as seen in the case described here. Moving forward, it will be essential to assess the outcomes and gather feedback from a broader group of learners to determine the effectiveness of using a Pb. Additionally, in the future, finding the right balance between maintaining the realism of the simulation will be essential. Overall, this case demonstrates how Pbs can strengthen education on cultural humility and support trainees in synthesizing their learning.

## AUTHOR CONTRIBUTIONS

Conceptualization: T. C., L. K., J. R., D. R., K. V.; Data curation: T. C., L. K., K. V.; Formal analysis: T. C., L. K., K. V., J. R., D. L.; Visualization: T. C.; Writing—original draft: T. C., L. K., K. V.; Writing—review and editing: T. C., L. K., J. R., D. R., K. V. The authors are accountable for all aspects of the work and provided final approval of the version to be published.

## Supporting information


Appendix S1

## Data Availability

The data that support the findings of this study are available on request from the corresponding author. The data are not publicly available due to privacy or ethical restrictions.
