# Supplementary material for: Implementing a prebrief for cultural humility in standardized patient sessions with genetic counseling students
Source: J Genet Couns. 2025 Aug 29;34(5):e70098. doi: 10.1002/jgc4.70098 (PMC12397680; doi:10.1002/jgc4.70098)
Supplement: Supplementary file 1 — Appendix S1 [file JGC4-34-0-s001.docx]

**Supplemental:**

**Implementation:**

While it does take thoughtful planning upfront, the use of a Pb before a GC session in this instance was easily incorporated into normal program operations. The length of the Pb was rather short and easily incorporated into the students’ Genetic Counseling and Theory classes, where they were already discussing their upcoming SP obligations in the curriculum. Given the relatively short time that a Pb takes, this could also be implemented online or asynchronously if a program is not able to incorporate it into an existing class. The Pb video or other materials, such as articles on the SP session topic could be sent to learners to watch on their own time and an online discussion board could be set up in lieu of an in-person discussion. Additionally, the Pb was added onto the training that the SPs all participated in to prepare them for the simulations with the learners. This SP training could also be implemented online if in person training is not feasible.

Our experience was fortunately in collaboration with the established and heavily resourced SP program at the University of Pennsylvania. The program has SP educators that established workflows to hire and train SPs, dedicated staff to help students on the day of the simulation, and technology to record the session in a simulated clinical environment. Computers are also provided for both learners and SPs to provide feedback and evaluations. While the case and Pb used here can be repeated at other programs, the logistics and feasibility of running the SP session and training the SPs could differ depending on how SPs are trained at other programs and if they have a dedicated SP educator.

SP Case Development

The case was created with input from genetic counselors who have seen similar cases. The case and the prebrief video shown to SPs and learners was developed by subject matter experts, including two Asian genetic counselors who provided more context and feedback regarding the topic of sex preference. The SP Program expanded upon background character details and standardized answers to counselor questions. As there were not enough SPs of Asian background, the simulation was written so that the topic of sex preference comes to light because the pregnant patient’s partner is Asian. This way, the cultural topic would be address without the SPs having to portray an ethnic background that they are not a part of.

SP Training

The SPs participating in the cases were all trained prior to the session. Training included role play, group discussion and practice of giving learner feedback. They viewed the same video that the genetic counseling learners watched surrounding the cultural context and significance behind the case. The SPs were additionally given an article that described the medical background and procedures that they were discussing in the case with the learners. Course directors also included an author commentary in the SP case from subject matter experts. Below is the full description of the case given to the SPs during training.

**Case Background:** You are Jenny Li, a 40-year-old paralegal. You and your husband live in Germantown. This is your first pregnancy. You and your husband, Cam, were married when you were both 30. He is a landscape architect and you met through a mutual friend. You wanted to be married for a couple years and continue establishing your career before starting a family. You are a paralegal with a patent law firm in Philadelphia (you’ve been working from home this past year). You and Cam have always thought you’d have 2 or 3 children. Cam has 3 brothers, and you have a brother and a sister. You have great relationships with your siblings and siblings-in-law. You’ve fantasized for years about having you and your children sitting around the dinner table playing games and having fun. You figured starting your family when you were 32 and having a baby every couple of years seemed like a good plan. That’s what your mom did, and you and your siblings are the best of friends. Your husband, Cam, is of Chinese descent and he is the oldest male in his family. Everyone is really hoping and wishing for a boy. This would mean so much to the family at large. You’ve always wanted a boy and if you’re only able to have one child, a boy would be your first choice. You got pregnant when you were 34 – it took a little longer to get pregnant than you thought it would. It was so exciting, you told everyone in your family right away, but you miscarried when you were 8 weeks pregnant, after you had taken a home pregnancy test, but before you had seen a doctor. Just days before your doctor appointment, but after home pregnancy tests. You and your husband felt so alone, so sad. You also felt shame at not being able to carry a pregnancy. It was very upsetting.
